# Construction and application of flow pressure drop model of perforated well considering pressure loss of perforation hole

**DOI:** 10.1038/s41598-023-46443-y

**Published:** 2023-11-04

**Authors:** Hongfeng Jiang, Muwang Wu, Yongjian Zheng, Qibin Zhao, Yongde Gao

**Affiliations:** 1 Zhanjiang Branch, CNOOC Limited (China), Zhanjiang, 524057 China; 2 CNOOC Limited (China), Beijing, 100010 China

**Keywords:** Crude oil, Petrol

## Abstract

Perforating well is one of the main production wells in reservoir development. Perforating effect directly affects well production, so the optimization of perforating parameters has attracted wide attention. Because pressure difference serves as the driving force for fluid flowing from formation to wellbore, it is important to understand the composition of production pressure difference in perforating well, which can guide the optimization of perforating parameters and the evaluation of perforating effect. In order to clarify the composition of production pressure difference during the production process of perforated wells, a pressure drop model pressure drop model is established based on fluid mechanics theory, which includes a pressure drop model of formation and a pressure drop model of perforation hole. The pressure drop model of formation is firstly constructed based on the Darcy's law and the equivalent resistance method, and the pressure drop model of perforation hole is built by the fluid tube-flow theory. Secondly, the numerical calculation method is adopted to realize the coupling solution of models, and the accuracy of this model is verified by comparison of the Karakas-Tariq model. Finally, the effects of formation physical properties and perforating parameters on flow pressure drop are discussed. The results show that there is a difference of more than 2 orders of magnitude between the pressure drop generated in perforation hole and flow pressure difference, and pressure drop of perforation hole can be neglected in practical applications. Comparing with medium–high permeability reservoirs, optimizing perforation parameters in low permeability reservoirs has a more significant impact on flow pressure drop. Among perforating parameters, perforation length and perforation density have great influence on flow pressure difference, while perforation diameter and phase angle have relatively little influence. These results have certain guiding significance for optimizing perforating parameters in different permeability reservoirs.

## Introduction

As the main means to establish the connecting channel between formation and wellbore, perforation technology is widely used in the development of reservoirs. Through the efforts of petroleum industry for many years, perforation technology has been continuously improved. A set of perforation technology has been formed, which can basically meet the requirements of reservoir development^1^. So far, perforation technology has gone through five development stages. The first stage is the bullet perforating technology in 1940s, which is complicated and inefficient. The second stage is the conventional shaped charge perforating technology in 1960s, which can significantly improve perforating efficiency. The third stage is the shaped energy enhanced perforation technology in 1980s, and this technology can greatly improve the perforation efficiency by scouring perforated hole and creating micro-cracks around perforated hole^2^. In the fourth stage, new perforating technologies emerge at the beginning of this century, which include dynamic negative pressure perforating technology, self-cleaning perforating technology and ultra-high perforation density, and they further improve the communication channel between formation and wellbore^3–6^. The fifth stage is the multi-stage perforation technology, which produces in recent years with the advancement of fracturing technology^7,8^. These advances of perforation techniques have provided some reliable methods for establishing efficient flow channels. Due to the diversity of reservoir types and the complexity of engineering technology, it is necessary to select appropriate perforation techniques based on the optimization results of perforation parameters.

**Figure 1:**
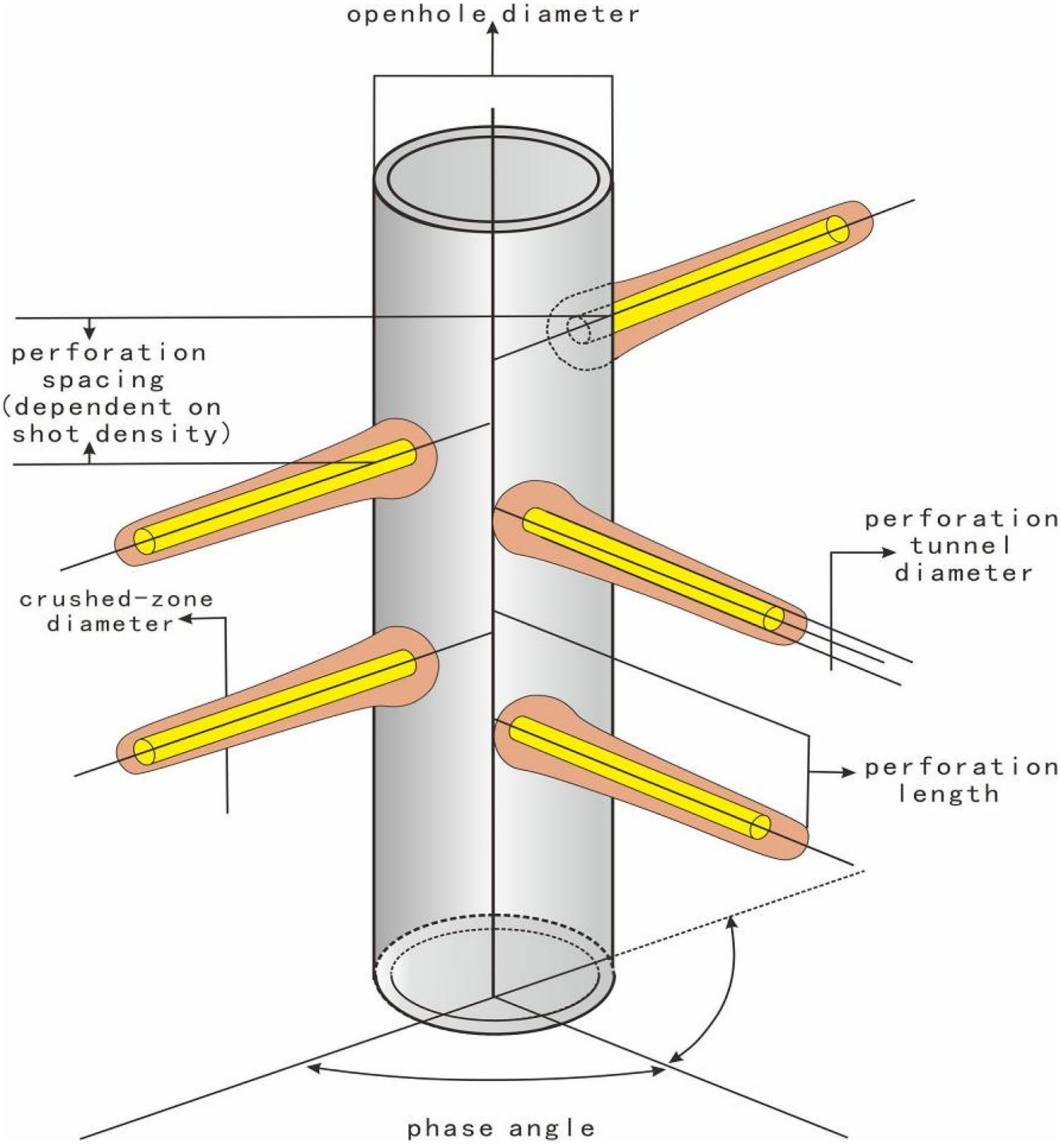
Reservoir perforation and completion diagram.

Reasonable perforation parameters can greatly relieve formation damage and significantly increase well production. Otherwise, unreasonable perforation parameters can cause obvious formation damage, and further reduce well productivity. Perforation parameters generally include hole aperture, hole density, hole length and phase angle (Fig. 1), which can determine the difficulty of formation fluid flowing into wellbore. Many scholars have conducted some studies on optimizing perforation parameters^9–12^. The optimization of perforation parameters is generally to build a perforation skin coefficient model or a production model of perforating well, which can determine perforation parameters by analyzing the relationship curves between perforation parameters and perforation skin or well production. In perforating well, perforation skin coefficient can represent the quality of perforating. The better perforating effect is, the smaller perforation skin coefficient is. According to reservoir physical characteristics and fluid flow characteristics around perforation hole, perforation skin is decomposed into three parts: the pseudo-surface coefficient generated by plane flow effect, the pseudo-skin coefficient generated by vertical flow effect, and the pseudo-skin coefficient generated by borehole effect. By constructing a relationship model between perforating parameters and perforation skin coefficient, the relationship curve between perforation skin coefficient and perforation parameters is obtained to determine the reasonable perforation parameters^13^. Karakas and Tariq^14^ builds a perforation skin prediction model under ideal conditions. Other studies consider the relationship between reservoir damage depth and hole depth, and build some skin coefficient prediction models adapted to different engineering conditions^15,16^. Sun et al.^17^ uses the Computational Fluid Dynamics Software to simulate flow process and calculates perforation skin under three-dimensional formation conditions, and compares with those calculated by Karakas-Tariq model, which shows that perforating optimization can be realized using the Computational Fluid Dynamics Software. Based on well production maximization to optimize perforating parameters, Li et al.^18^ applies the principle of seepage mechanics and the principle of equivalent resistance to give a productivity equation of partial perforating well in isotropic reservoir, and discusses the relationship between well productivity and perforation parameters. Considering reservoir heterogeneity and well type variability, Wang et al.^19^ builds a optimization model of perforation parameters to maximize production, and proposes a perforation parameters optimization method for horizontal wells. Some scholars realize the optimization of perforation parameters from some perspectives of liquid production profile optimization and casing safety^20–22^. These methods only optimize perforation parameters from the perspective of obtaining maximum production, which can’t explain for increasing production from flow mechanism. In reservoirs, fluid flows from the formation to the wellbore under pressure difference, so perforation parameters can be optimized through the composition of pressure drops in various parts during the production process of perforated well.

In addition, there is no analysis on the pressure drop in perforation hole of perforating well, but pressure drop is obvious in flow process of long-distance horizontal well^23–26^. When fluid flows in horizontal well, there is friction between wellbore and fluid, which results in pressure loss. Dikken et al.^27^ establishes a well productivity model considering wellbore friction loss of horizontal well, and proves that friction loss of wellbore has a certain influence on well productivity. Ihara et al.^28^ considers the pressure drop caused by flow friction and fluid mobility, and analyzes the influence of wellbore inflow on the pressure drop using single phase rectangular tube experiment. By comparing the results of physical simulation experiments, Schulks et al.^29^ considers that fluid injection has a certain lubricating effect on wall flow when wall flow velocity is less than mainstream velocity. While wall flow velocity is greater than mainstream velocity, fluid injection obstructs fluid flow in wellbore and increases pressure drop of wellbore, which indicates the influence of fluid flow form on pressure drop^29^. In addition, some relevant scholars adopt various methods to analyze the influence of multiphase flow, fluid and channel parameters on pressure drop^30,31^. Fluid flow of perforated tunnel is similar to that of horizontal wellbore, so it is necessary to analyze pressure drop in perforation hole.

In this paper, flow pressure drop of perforated well is studied. Firstly, fluid flow of formation is divided into three zones, which contains radial flow in reservoir zone, radial flow through uncrushed zone and radial flow through crushed zone, and a pressure drop model of fluid flow is constructed using equivalent resistance method. Simultaneously, a pressure drop model of perforation hole is built on the fluid tube flow theory. Then, the coupling solution of these models is realized by using numerical calculation method, and the accuracy of this model is verified by comparing with the results of Karakas-Tariq model. Finally, the effects of formation physical properties and perforating parameters on pressure drop are discussed based on this model. These results have certain guiding significance to the optimization of perforation parameters.

## Mathematical model of fluid flow

Perforation is a main means to establish the flow channel between formation and wellbore, which is widely used in the development of oil and gas reservoirs. During the production of perforation well, fluid flows from formation through perforated hole into wellbore. Therefore, fluid flow mainly consists of the flow of formation and perforation hole. It is assumed that fluid is single-phase fluid, the flow of formation obeys the Darcy's law, and the flow of perforation hole obeys the law of tube-flow.

### Fluid flow model in reservoir

**Figure 2:**
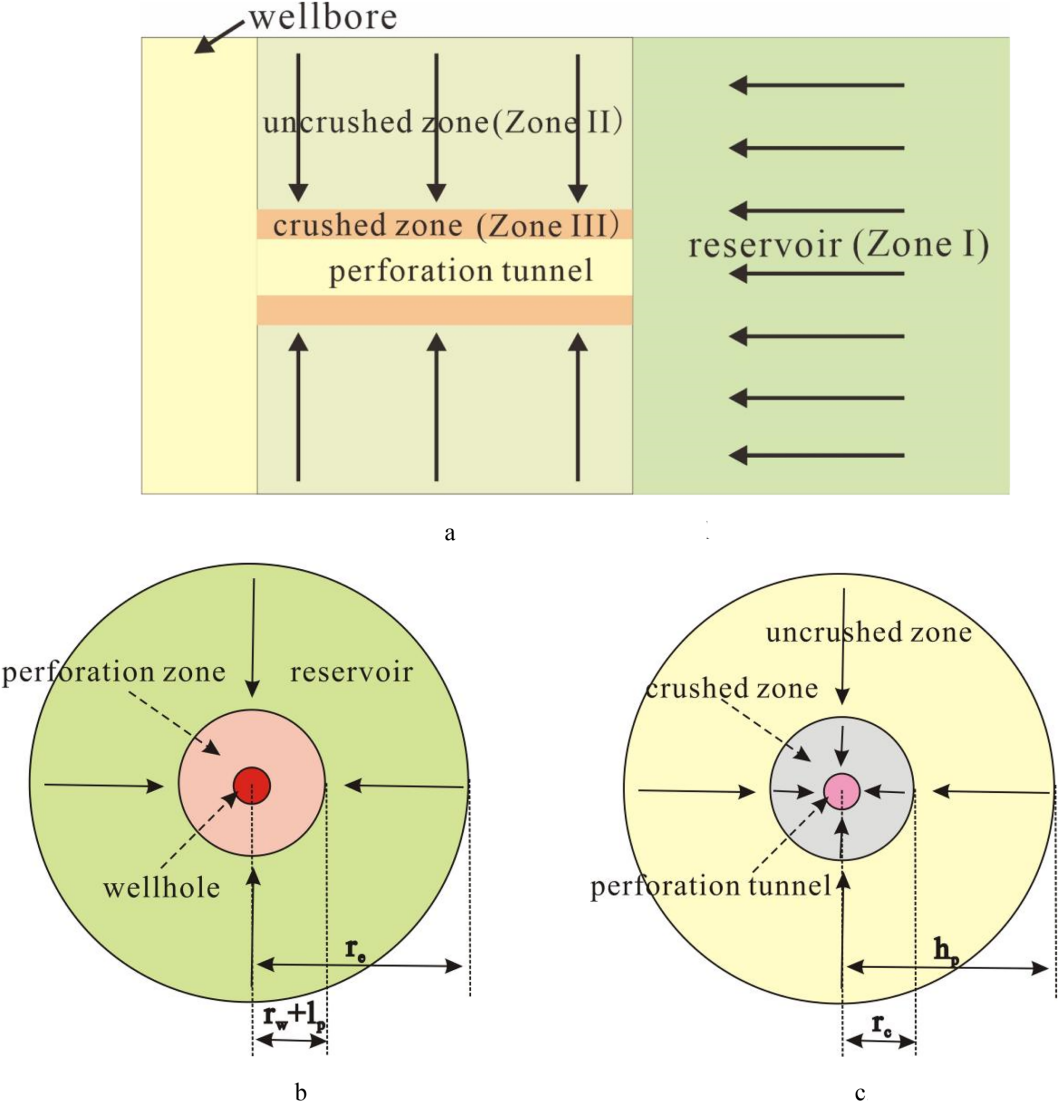
Three radial flow model of perforated well. (**a**) Three radial flow model. (**b**) Radial flow model in reservoir area. (**c**) Radial flow model in perforated area.

During ideal formation conditions, fluid flows from formation to perforation hole mainly through reservoir zone and perforated zone. Considering that there is a obvious crushed zone near perforation hole, flow from reservoir to perforation hole can be simplified into a three-radial flow process. It includes the radial flow during the reservoir zone (Zone I), the radial flow during the uncrushed zone (Zone II), and the radial flow during the crushed zone (Zone III) (Fig. 2a).

In reservoirs (zone I), if there is a constant pressure boundary and formation fluid flows stably (Fig. 2b), the well production equation can be written as:

1 $$ q_{I} = J_{I} [p_{e} - p(r)] $$

Under the condition of stable radial flow, the index of oil production is:

2 $$ J_{I} = \frac{{2\pi k_{{\text{I}}} h}}{{\mu \ln \frac{{r_{e} }}{r}}} $$

The pressure drop generated by the fluid flowing in zone I is:

3 $$ \Delta p_{I} = \frac{q\mu }{{2\pi k_{I} h}}\ln \frac{{r_{e} }}{r} $$

In the uncrushed region (zone II), fluid flows radially around the vertical plane of hole (Fig. 2c), and its production can be expressed as:

4 $$ q_{II} = J_{II} [p(r) - p(r_{c} )] $$

Under the condition of stable radial flow, the oil production index of Zone II can be expressed as:

5 $$ J_{II} = \frac{{2\pi k_{II} l_{p} }}{{\mu \ln \frac{{h_{p} }}{{r_{c} }}}} $$

where *h*_*p*_ is the distance between the same phase angle of two adjacent hole, $h_{p} = 180/n_{s} /\theta$.

The pressure drop generates by the fluid flowing in the zone II is:

6 $$ \Delta p_{II} = \frac{{q\mu \ln \frac{{h_{p} }}{{r_{c} }}}}{{2\pi k_{II} l_{p} }} $$

In the crushed zone (zone III), the flow pattern is similar to that in Zone II, and its production can be expressed as:

7 $$ q_{III} = J_{III} [p(r_{c} ) - p(r_{p} )] $$

Under the condition of stable radial flow, the oil production index of Zone III can be expressed as:

8 $$ J_{III} = \frac{{2\pi k_{III} l_{p} }}{{\mu \ln \frac{{r_{c} }}{{r_{p} }}}} $$

The pressure drop of zone III is:

9 $$ \Delta p_{III} = \frac{{q_{III} \mu \ln \frac{{r_{c} }}{{r_{p} }}}}{{2\pi k_{III} l_{p} }} $$

During the steady flow of fluid from the formation to the perforation hole, the equivalent resistance method can be used to obtain the pressure drop. It is:

10 $$ \Delta p_{R} = p_{{_{e} }} - p(r_{p} ) = \Delta p_{I} + \Delta p_{II} + \Delta p_{III} $$

### Fluid flow model in perforation hole

**Figure 3:**
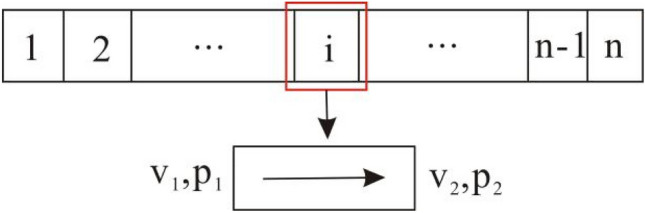
Schematic diagram of fluid flow in the perforation hole.

Fluid flowing in perforation hole can be regarded as the composition of multiple units, and the flow of each unit is similar to the flow of horizontal wellbore. Assuming that the length of micro-unit is Δx, the upstream pressure of micro-unit is *p*_*1*_ and the downstream pressure is *p*_*2*_. The pressure loss of fluid flow is mainly caused by the friction resistance between fluid and tube, and the friction resistance within the fluid^32^ (Fig. 3). In this unit, the momentum equation of fluid is^32^:

11 $$ p_{1} A - p_{2} A - \tau \pi D\Delta x = \rho Q_{2} v_{2} - \rho Q_{1} v_{1} $$

The fluid mass flow rate mainly includes the upper fluid mass flow rate and the fluid inflow of this unit, and the continuity equation can be expressed as:

12 $$ Av_{1} + q = Av_{2} $$

Without the influence of heat transfer, its energy equation can be written as^33^:

13 $$ \frac{{p_{1} }}{\rho g} + \frac{{v_{1}^{2} }}{2g} = \frac{{p_{2} }}{\rho g} + \frac{{v_{2}^{2} }}{2g} + \frac{{f\overline{v}^{2} }}{2Dg}\Delta x + h_{12} $$

The average velocity in the unit can be expressed as:

14 $$ \overline{v} = \frac{{v_{1} + v_{2} }}{2} $$

When fluid flows in perforation hole, the friction coefficient between fluid and hole wall is related to the flow law. In the laminar flow, the friction coefficient is as follows^33^:

15 $$ f = \frac{64}{{N_{{R_{e} }} }} $$

The friction coefficient in the turbulent flow is^33^:

16 $$ 4f = [1.14 - 2\log (\varepsilon /D + 21.25N_{{\text{Re}}}^{ - 0.9} )]^{2} $$

Equation (13) is applied for 4000 ≤ *N*_*Re*_ ≤ 10^8^ and 10^–8^ ≤ *ε/D* ≤ 0.1, *N*_*Re*_ is the Reynolds number:

17 $$ N_{{R_{e} }} = \rho vd/\mu $$

Assuming the perforation hole is horizontal, the pressure drop generated in perforation hole can be written as:

18 $$ p_{1} - p_{2} = \frac{\rho }{2}(v_{2}^{2} - v_{1}^{2} ) + \frac{{f\rho \overline{v}^{2} }}{2D}\Delta x $$

## Solution of flow model

Since there is only a single phase fluid flow in formation and perforation hole, the pressure drop of fluid flowing from formation into wellbore mainly includes the pressure drop of reservoir and perforation hole. Therefore, the pressure drop formula of formation fluid flowing into wellbore can be derived. The perforation hole is divided into N micro-units (Fig. 3), and the pressure drop of fluid flowing into the i-th unit from the formation can be expressed as:

19 $$ \Delta p(r_{p,i} ) = \frac{{q\mu \ln \frac{{r_{e} }}{r}}}{{2\pi k_{I} h}} + \frac{{q_{i} \mu \ln \frac{{h_{p} }}{{r_{c} }}}}{{2\pi k_{II} l_{p} }} + \frac{{q_{i} \mu \ln \frac{{r_{c} }}{{r_{p} }}}}{{2\pi k_{III} l_{p} }} $$

The pressure drop generates from the i-th micro-unit into the wellbore, it is:

20 $$ \Delta p_{p,i} = \frac{\rho }{2}(v_{i,2}^{2} - v_{i,1}^{2} ) - \frac{{f\rho \overline{v}_{i}^{2} }}{2D}x_{i} $$

Based on the superposition principle of pressure drop, the pressure drop of reservoir can be built when M holes in formation are produced. It is:

21 $$ \Delta p_{R} = q\frac{{2\pi k_{I} h}}{{\mu \ln \frac{{r_{e} }}{r}}} + \frac{q}{M}\left( {\frac{{2\pi k_{II} l_{p} }}{{\mu \ln \frac{{h_{p} }}{{r_{c} }}}} + \frac{{2\pi k_{III} l_{p} }}{{\mu \ln \frac{{r_{c} }}{{r_{p} }}}}} \right) $$

Similarly, the pressure drop generates in perforation hole is:

22 $$ \Delta p_{p} = \sum\limits_{i = 1}^{N} {\Delta p_{p,i} } = \sum\limits_{i = 1}^{N} {\left[ {\frac{\rho }{2}(v_{i,2}^{2} - v_{i,1}^{2} ) - \frac{{f\rho \overline{v}_{i}^{2} }}{2D}x_{i} } \right]} $$

Therefore, the total pressure drop from formation into wellbore can be expressed as:

23 $$ \Delta p = \Delta p_{R} + \Delta p_{p} $$

If the production is equal for each perforation hole, the total production of well can be expressed as:

24 $$ q = M\sum\limits_{i = 1}^{N} {q_{i} } $$

A matrix equation of (N + 1) × (N + 1) can be formed according to the pressure drop equation and the total production equation. Firstly, the output of each unit is given, the hole friction coefficient is calculated, and the solving coefficient of matrix equations is determined. Then, the bottom hole pressure and the output of each hole unit can be obtained by solving the matrix equation. The production of each hole unit is calculated to decide the hole friction coefficient, and the coefficient of matrix equations is determined to solve the bottom hole pressure and the production. The bottom hole pressure and the production of each hole unit are calculated through several iterations, and the pressure drop of each flow area is calculated.

## Model verification

**Figure 4:**
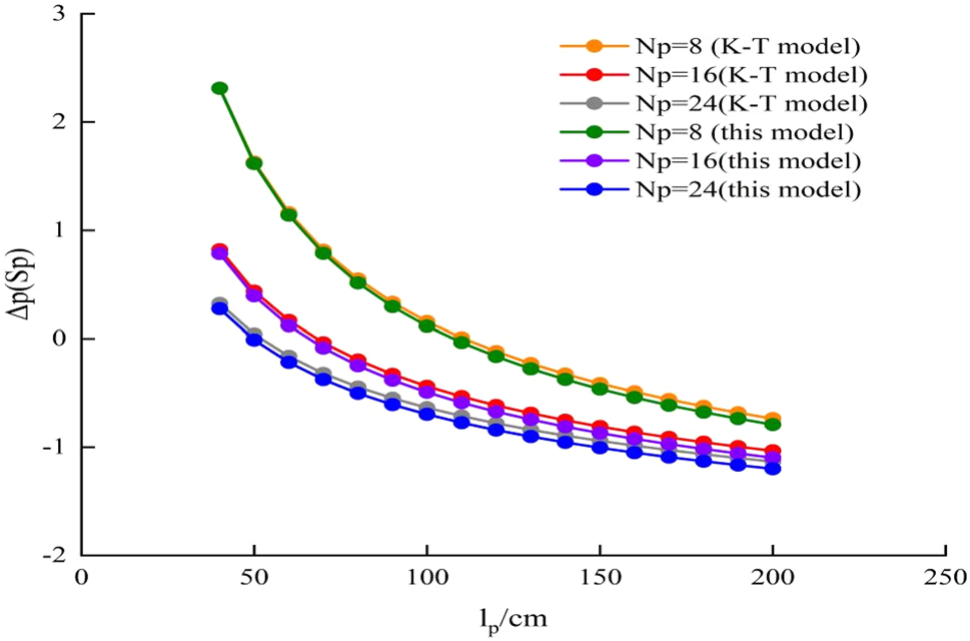
Comparison of calculation results between this model and K-T model.

The pressure drop model of perforating well can calculate the pressure difference of each zone, and the total pressure drop compares with that of an open hole completion condition at the same production, which can be used to evaluate the perforation effect. Karakas and Tariq proposes a calculation model (K-T model) of perforating skin coefficient^14^. Based on the K-T model, the perforating skin coefficient can be calculated, and the additional pressure drop generated by perforation can be obtained. Under the same conditions, the additional pressure generated by perforation can be obtained based on the pressure drop model. The accuracy of the two models can be verified by comparing the additional pressure drop. The values of relevant parameters are as follows: q = 100 m^3^/d, k_I_ = 100 mD, k_II_ = 45 mD, k_III_ = 10 mD, h = 10 m, μ = 2.5 mPa•s, r_e_ = 300 m, r_w_ = 10 cm, θ = 45°, r_p_ = 1.0 cm, r_c_ = 0.05 cm. The comparison results of these two models are shown in Table 1 and Fig. 4. The results show that the pressure drop calculated by these models is almost consistent. It indicates the reliability of the pressure drop model.

**Table 1:** Comparison of calculation results between this model and K-T model.

lp/cm	Δp(Sp)/MPa Np = 8			Δp(Sp)/MPa Np = 16			Δp(Sp)/MPa Np = 24		
	K–T model	This model	Error/%	K–T model	This model	Error/%	K–T model	This model	Error/%
50	1.63	1.62	0.61	0.44	0.40	9.09	0.04	– 0.01	–
100	0.16	0.12	25.00	– 0.44	– 0.49	11.36	– 0.64	– 0.70	9.37
150	– 0.41	– 0.46	12.20	– 0.81	– 0.87	7.41	– 0.94	– 1.01	7.45
200	– 0.74	– 0.79	6.76	– 1.03	– 1.10	6.80	– 1.13	– 1.20	6.19

## Analysis and discussion

During the production of well, production pressure difference is mainly controlled by geological and engineering factors. In order to understand the pressure difference generated by each zone of formation fluid flowing into wellbore, it assumes that the relevant parameters are the same as those in the model validation section. The influence of permeability, perforation parameters and production rate on the production pressure difference is analyzed.

(1) Pressure drop of perforation hole

**Figure 5:**
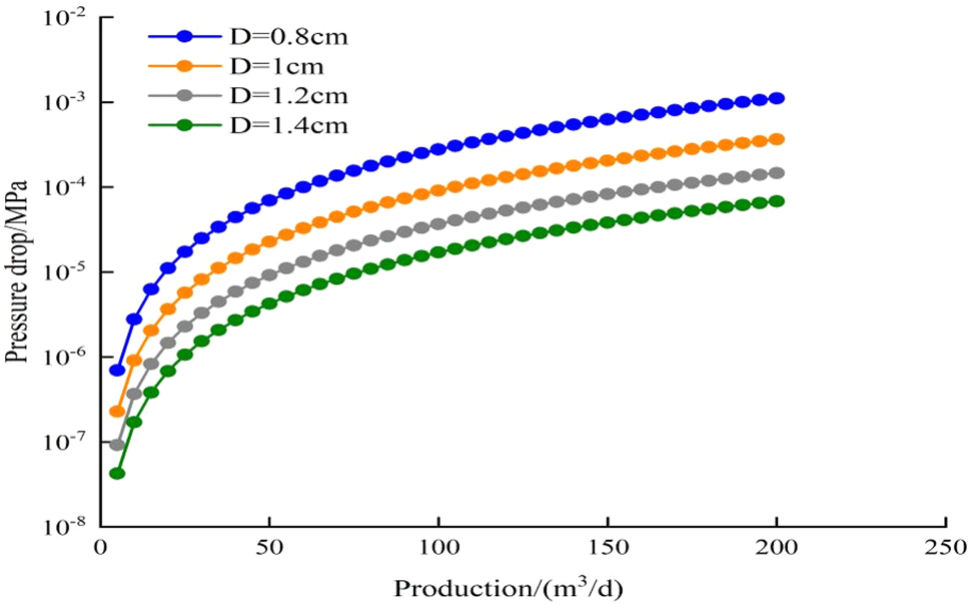
Relationship curve between pressure drop and production under different hole diameters.

**Figure 6:**
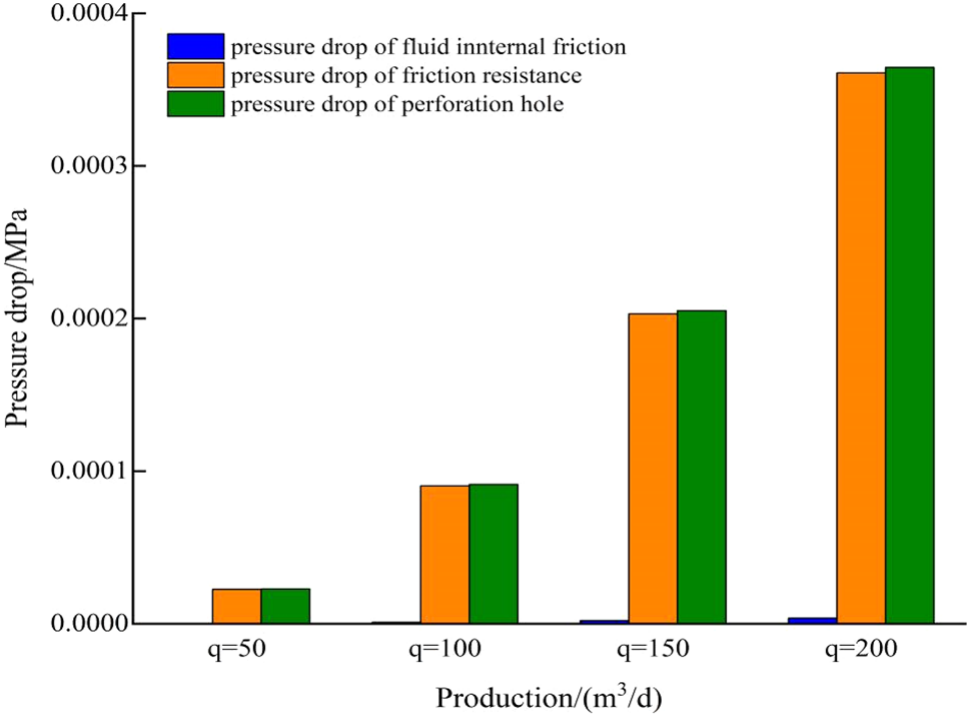
Composition of pressure drop in the hole under different production.

When fluid flows from formation into perforation hole, the flow pattern changes from Darcy flow to tube flow. According to the principle of tube flow, the pressure drop in perforation hole includes the pressure drop caused by internal friction between fluid and tube and the pressure drop of friction resistance. If the length of perforation hole is 100 cm, the hole density is 16 holes/m, the phase angle is 45°, and the friction coefficient is 0.5. The model is used to calculate the relationship between the pressure drop of perforating hole and production under different hole diameter (Fig. 5). When well production increases from 5 to 200 m^3^/d, the flow velocity and flow pressure drop caused by friction resistance increase obviously, but its maximum value is only 0.0036 MPa. Because the diameter of perforating hole directly affects the flow velocity, the diameter of hole has an obvious influence on the pressure drop. The pressure drop of perforation hole mainly comes from the friction between fluid and hole, which accounts for more than 90% of the total pressure drop (Fig. 6). Fluid flow from reservoir to well bottom includes the pressure drop of perforating hole, zone I, Zone II and zone III. The calculated results of each part pressure drop under different production rates are shown in Table 2. The difference between the pressure drop of perforating hole and the total pressure drop is more than 2 orders of magnitude. In practical engineering calculation, the pressure drop of perforating hole can be ignored.

**Table 2:** Pressure drop calculation results of each part under different production.

q (m^3^/d)	Δp_p_/MPa	Δp_I_/MPa	Δp_II_/MPa	Δp_III_/MPa	Δp/MPa
q = 50	0.00002	1.29	0.05	0.33	1.67
q = 100	0.00009	2.58	0.10	0.66	3.35
q = 150	0.00021	3.87	0.15	0.99	5.02
q = 200	0.00036	5.17	0.21	1.33	6.70

(2) Influence of formation permeability

**Figure 7:**
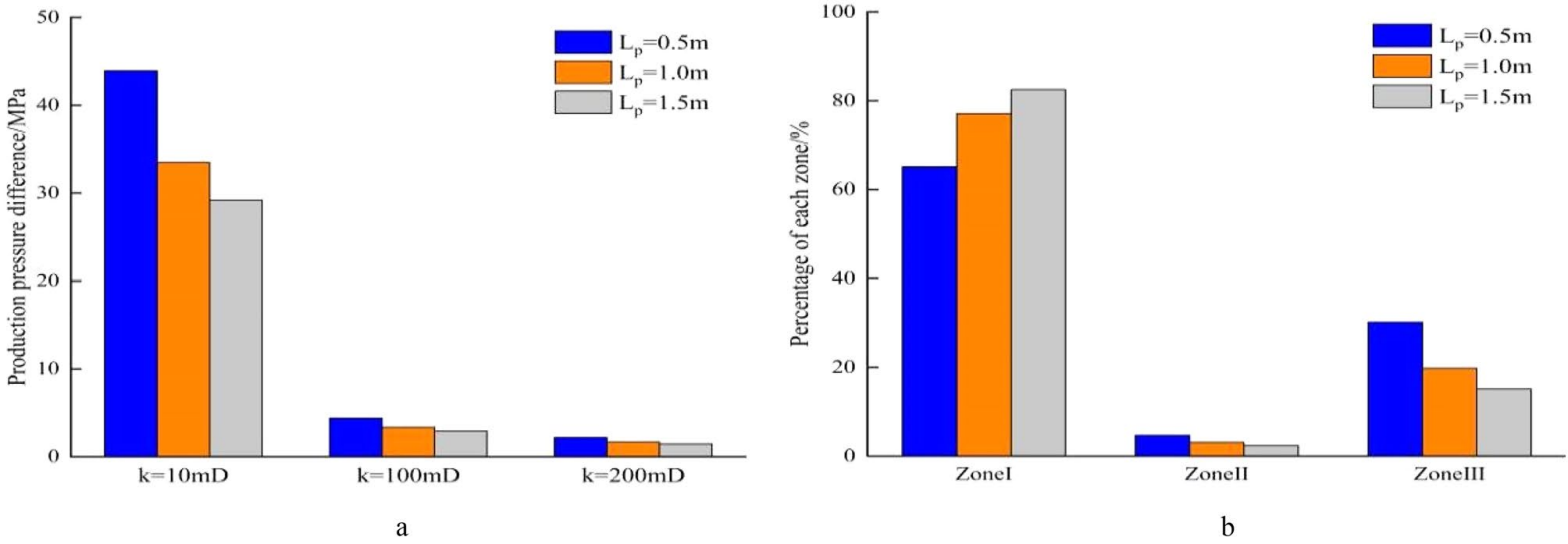
Composition of production pressure drop under different hole lengths. (**a**) Chart of production pressure difference. (**b**) Pressure drop percentage of each zone.

**Figure 8:**
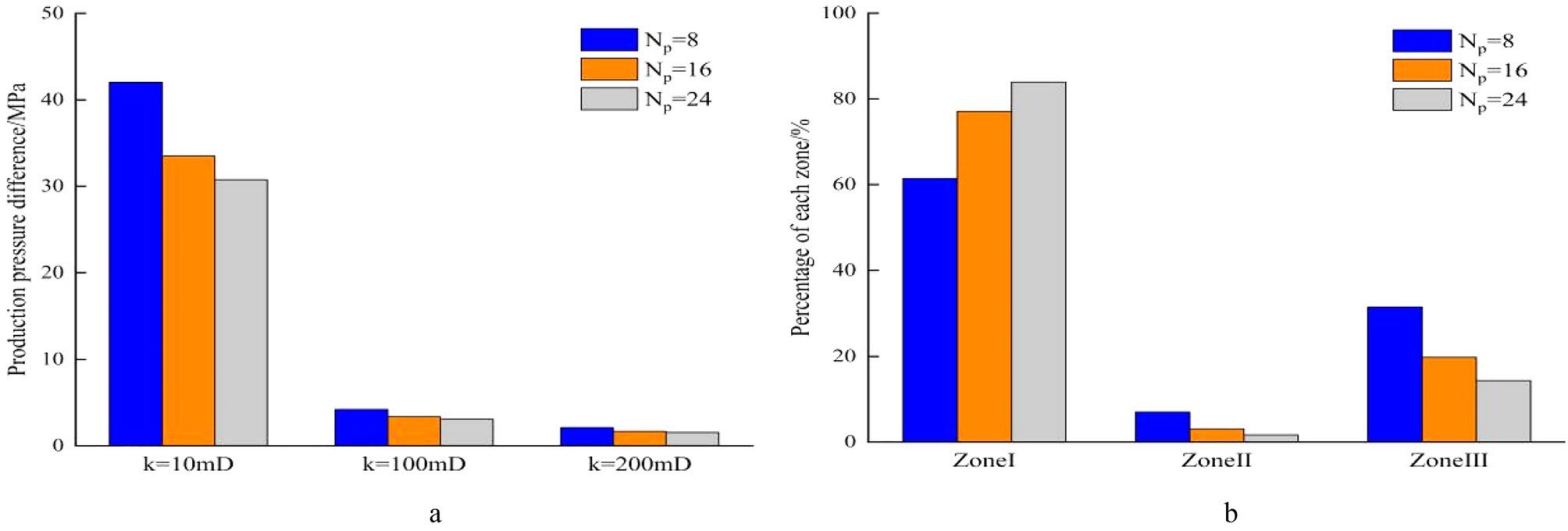
Composition of production pressure difference under different hole densities. (**a**) Chart of production pressure difference. (**b**) Pressure drop percentage of each zone.

**Figure 9:**
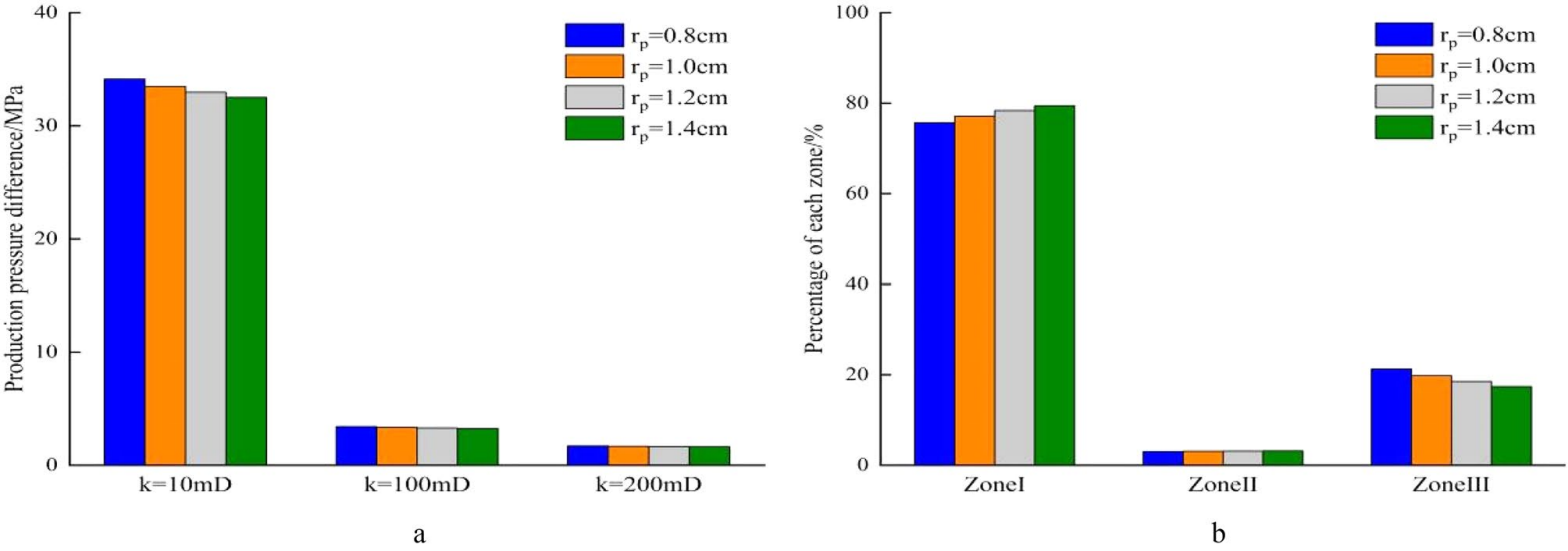
Composition of production pressure difference under different hole diameters. (**a**) Chart of production pressure difference. (**b**) Pressure drop percentage of each zone.

**Figure 10:**
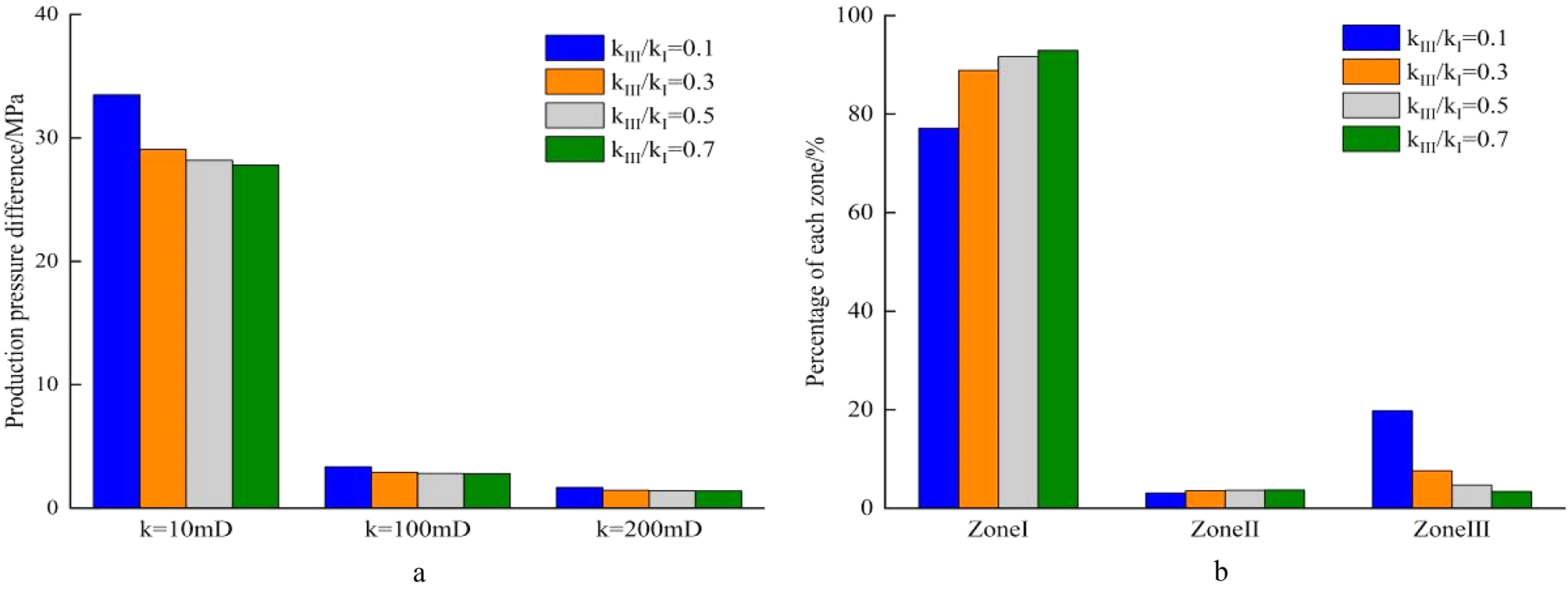
Composition of production pressure difference under different permeability of crushed zone. (**a**) Chart of production pressure difference, (**b**) pressure drop percentage of each zone.

During the development of homogeneous reservoirs, the permeability can represent formation physical property. The production pressure difference is the sum of pressure drops of fluid flowing from the formation to the well bottom. When well production is 100m^3^/d, the length of perforation hole increases, the flow resistance near well decreases, and the pressure drop in reservoir decreases (Fig. 7a). In high permeability reservoirs, the production pressure difference is much smaller than that in low permeability reservoirs. Increasing perforation length can significantly reduce the percentage of flow resistance in perforation area, and improves the flow efficiency (Fig. 7b). Similarly, an increase of perforating density leads to decrease in the pressure drop of perforating area. In the reservoir with the permeability of 10mD, the production pressure difference drops from 42.03 to 30.75 MPa when the perforating density increases from 8pots/m to 24pots/m. In the reservoir with the permeability of 200mD, the production pressure difference decreases by only 0.56 MPa as the perforation density increases from 8 pots/m to 24 pots/m (Fig. 8a). During the process of increasing perforation density, the percentage of pressure drop increases in Zone I and significantly decreases in Zone III (Fig. 8b). When the diameter of perforation hole increases from 0.8 to 1.4 cm, the production pressure difference in the different permeability of reservoir decreases slightly, and the percentage of pressure drop in the three regions changes little (Fig. 9a,b). Due to the development of perforating techniques, some new perforating techniques can significantly improve the permeability of crushed zone, and the influence of permeability in the crushed zone is discussed here. As *k*_*III*_/*k*_*I*_ increases from 0.1 to 0.7, the production pressure difference decreases from 33.48 to 27.8 MPa in the reservoir with *k*_*I*_ = 10mD, while the production pressure difference decreases from 33.48 to 29.1 MPa when *k*_*III*_/*k*_*I*_ increases from 0.1 to 0.3. The production pressure difference only decreases from 1.67 to 1.39 MPa in the reservoir with *k*_*I*_ = 200 mD (Fig. 10a). The same law is also reflected in the percentage of pressure drop in these three regions. When *k*_*III*_/*k*_*I*_ increases from 0.1 to 0.3, the percentage of pressure drop in Zone I obviously increases, Zone III obviously decreases, and Zone II has little change. However, when *k*_*III*_/*k*_*I*_ increases from 0.3 to 0.7, the pressure drop percentage does not change significantly (Fig. 10b). During the perforating process, the permeability of crushed zone is too small, which may have a great impact on the productivity. When it increases to a certain extent, the flow resistance is limited. Therefore, it is not necessary to pursue the permeability of crushed zone during the perforating process. Because the production pressure difference in medium and high permeability reservoirs is much smaller than that in low permeability reservoirs, the optimization of perforation parameters can reduce the production pressure difference to a certain extent, but it is not as obvious as that in low permeability reservoirs. Therefore, more attention should be paid to the optimization of perforation parameters in low permeability reservoirs.

(3) Influence of perforation parameters

**Figure 11:**
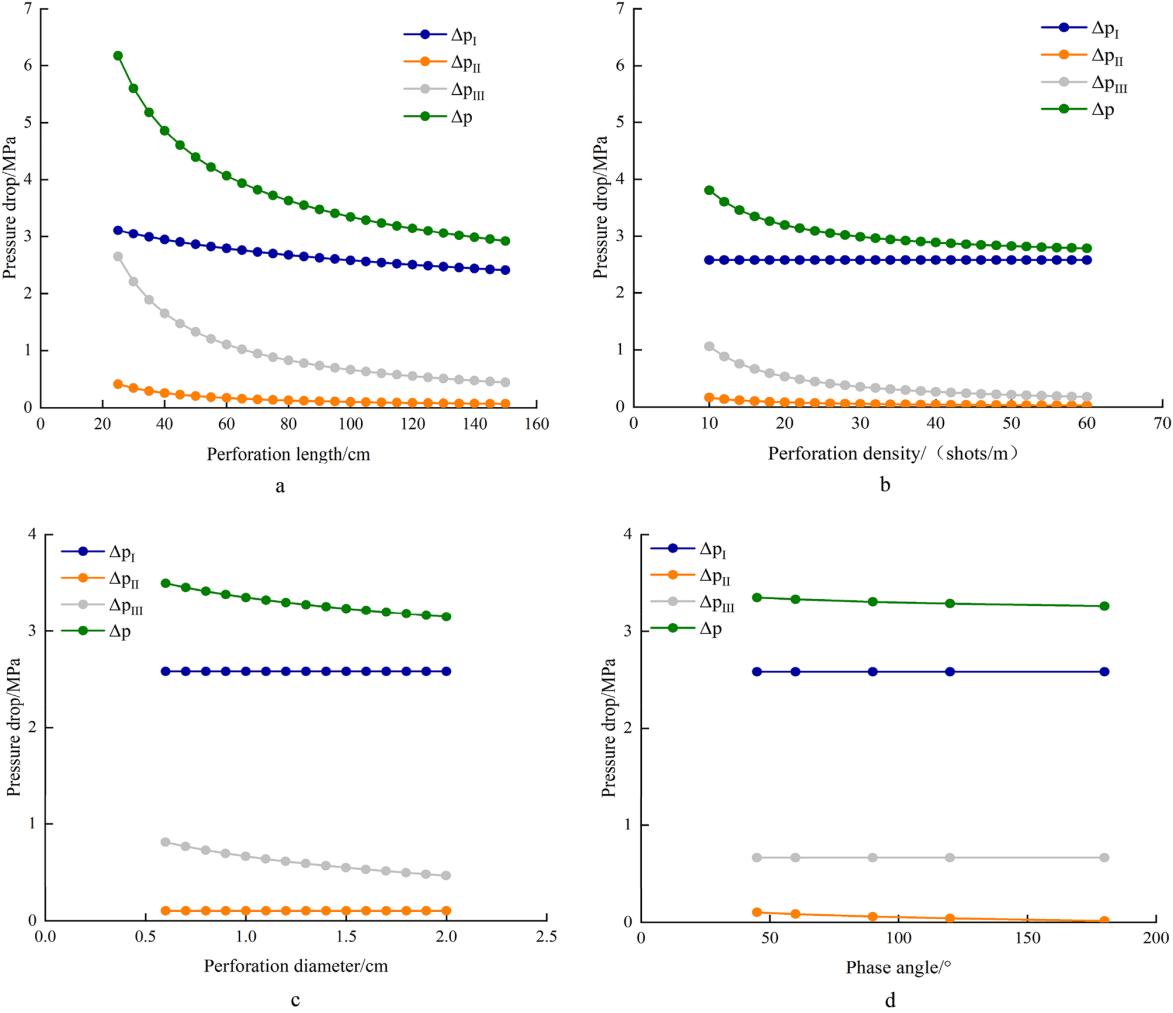
Pressure drop curves under different perforating parameters. (**a**) Pressure drop curves under different hole lengths. (**b**) Pressure drop curves under different hole densities. (**c**) Pressure drop curves under different hole diameters d Pressure drop curves under different phase angles.

Perforation is a key means to establish the communication channel between formation and wellbore. Perforation parameters should be designed during the process of perforation, which mainly include perforation length, perforation density, perforation diameter and phase angle. Perforation length determines the size of perforated zone. The increase of perforation length expands the range of perforation area, which helps to reduce the pressure drop when fluid flows in the perforation zone. Figure 11a shows the relationship between pressure drop and perforation length in each zone. As the hole length increases, the pressure drop of Zone I and zone II decreases slowly. The rapid change in pressure drop of Zone III is due to the fast flow rate of fluid around the near perforation and the wellbore, which leads to further interaction between the flow and the formation. As the hole density increases, the size of perforation area does not change, and the pressure drop of reservoir zone does not change. In the perforation zone, the distance of fluid entering the perforation hole becomes smaller, and the pressure drop decreases. With the increase of perforation density, the pressure drop of Zone II and Zone III decreases obviously when the perforation density is little, and gradually weakens when the perforation density becomes large (Fig. 11b). When the perforation diameter increases, the pressure drop of Zone I and Zone II is basically unchanged, while the pressure drop of Zone III decreases to a certain extent (Fig. 11c). When the phase angle increases, the pressure drop of Zone I and Zone III is basically unchanged, and the pressure drop of Zone II is slightly decreased (Fig. 11-d). Therefore, the perforation length and perforation density have greatly influence on pressure difference among these perforation parameters, while perforation diameter and phase angle have relatively little influence. During the optimization of perforation parameters, the optimization of perforation length and perforation density should be paid more attention.

## Conclusions

1. Considering the pressure loss of perforated hole and formation, the flow pressure drop models of seepage zone and tube-flow zone are constructed, which are based on the equivalent resistance method and the tube-flow theory. The coupled solution of model is realized by numerical calculation method, and the accuracy of this model is verified by comparing with the existing model.
2. The flow pressure drop of perforation hole is more than 2 orders of magnitude different from the production pressure difference, so the pressure drop of perforation hole can be ignored.
3. The production pressure difference in medium–high permeability reservoirs is much smaller than that in low permeability reservoirs. The optimization of perforation parameters can reduce the production pressure difference to a certain extent, but it is not as obvious as that in low-permeability reservoirs. Optimization of perforation parameters is more important in low permeability reservoirs.
4. In perforation parameters, perforation length and perforation density have great influence on the production pressure difference, while perforation diameter and phase angle have relatively little influence. During the optimization of perforation parameters, the optimization of hole length and hole density should be focused on.

## Data Availability

The datasets used and/or analysed during the current study available from the corresponding author on reasonable request.

## Abbreviations

*A*: Cross-sectional area of perforation hole (m^2^)
*D*: Diameter of perforation hole (m)
*d*: Characteristic length of hole (m)
*f*: Friction resistance coefficient
*g*: Gravitational acceleration (m^2^/s)
*h*: Formation thickness (m)
*h*_*p*_: Distance between the same phase angle of two adjacent hole (m)
*h*_*12*_: Elevation difference (m)
I: Reservoir zone
II: Uncrushed zone
III: Crushed zone
*J*: Oil production index (m^3^/(d MPa))
*k*: Permeability (mD)
*l*_*p*_: Depth of perforating hole (m)
*N*_*Re*_: Reynolds number
*n*_*s*_: Perforation density (pot/m)
*p*_*e*_: Pressure at the outer boundary of reservoir (MPa)
*p(r)*: Pressure at the outer boundary of perforating zone (MPa)
*p(r*_*c*_*)*: Formation pressure at the outer boundary of crushed zone (MPa)
*p*_*(*_*r*_*p)*_: Pressure of perforating hole (MPa)
*Q*: Production of perforating hole (m^3^/d)
*q*: Well production (m^3^/d)
*r*: Radius of perforating area (m)
*r*_*c*_: The radius of crushed zone (m)
*r*_*e*_: Radius of outer boundary (m)
*r*_*p*_: Radius of perforating hole (m)
*r*_*w*_: Well radius (m)
*v*: Velocity (m/s)
*μ*: Viscosity of crude oil (mPa s)
*τ*: Shear stress (N/m^2^)
*ρ*: Fluid density (kg/m^3^)
*ε*: Roughness coefficient (mm)
*θ*: Phase angle (°)
$\overline{v}$: Average flow rate (m/s)
*Δp*: Pressure drop (MPa)
*Δp*_*p*_: Pressure drop of perforation hole (MPa)
*Δp*_*R*_: Pressure drop in reservoir (MPa)
*Δp(r*_*p,i*_*)*: Pressure drop from formation into i-th unit (MPa)
*Δp*_*p*__*,i*_: Pressure drop from the i-th micro-unit into the wellbore (MPa)
*Δx*: Unit length (m)
